# Vascular Cognitive Impairment—The Molecular Basis and Potential Influence of the Gut Microbiota on the Pathological Process

**DOI:** 10.3390/cells13231962

**Published:** 2024-11-27

**Authors:** Piotr Olejnik, Aleksandra Golenia

**Affiliations:** Department of Neurology, Medical University of Warsaw, 02-091 Warsaw, Poland; aleksandra.golenia@wum.edu.pl

**Keywords:** vascular cognitive impairment, vascular dementia, molecular mechanism, gut microbiota

## Abstract

Cognitive impairment is a major healthcare challenge worldwide, with vascular cognitive impairment (VCI) being its second leading cause after Alzheimer’s disease. VCI is a heterogeneous group of cognitive disorders resulting from various vascular pathologies. Therefore, it is particularly difficult to determine its underlying cause and exact molecular basis. Nevertheless, the current understanding of the pathophysiological processes underlying VCI has changed and evolved in the last decades. The aim of this narrative review is to summarize the current state of knowledge on VCI pathogenesis and to analyze the potential role of the gut microbiota in this process, considering the most recent scientific reports and in accordance with the current understanding of these processes. Chronic cerebral hypoperfusion, which results in impaired blood supply, i.e., oxygen and nutrient deficiency, is the main underlying mechanism of VCI. Furthermore, chronic cerebral hypoperfusion triggers a cascade of molecular changes, starting with an energy imbalance, leading to glutamate excitotoxicity, acidotoxicity, and oxidative stress. Also, all of the above provoke the activation of microglia and the release of pro-inflammatory cytokines that recruit systemic immune cells and lead to their infiltration into the central nervous system, resulting in neuroinflammation. Blood–brain barrier dysfunction may occur at various stages of chronic cerebral hypoperfusion, ultimately increasing its permeability and allowing potentially toxic substances to enter the brain parenchyma. Gut microbiota and their metabolites, which have been identified in numerous inflammatory conditions, may also influence the pathophysiological processes of VCI.

## 1. Introduction

As the global population continues to age, cognitive impairment is becoming a major healthcare challenge worldwide [1], and vascular cognitive impairment (VCI) is thought to be the second most common cause of dementia after Alzheimer’s disease [2]. Currently, VCI is a growing area of concern that requires greater attention in policy, healthcare, and research [3]. The prevalence of VCI may still be underestimated, as it shows considerable geographical variation. It ranges from approximately 20% of all dementia cases in Europe and North America to up to 30% in Asia and developing countries [4]. Hence, genetic predispositions, socioeconomic deprivation, and unhealthy lifestyle factors are well-established contributors that can increase the risk of developing dementia [5]. Individuals with lower socioeconomic status not only exhibit significantly higher risk of dementia [6], but also have an elevated prevalence of cardio-cerebrovascular diseases, which are among the greatest risk factors for VCI [7]. Additionally, the incidence of VCI rises with age, contributing significantly to the rising global prevalence of dementia, which is expected to nearly triple by 2050 [8].

**Figure 1:**
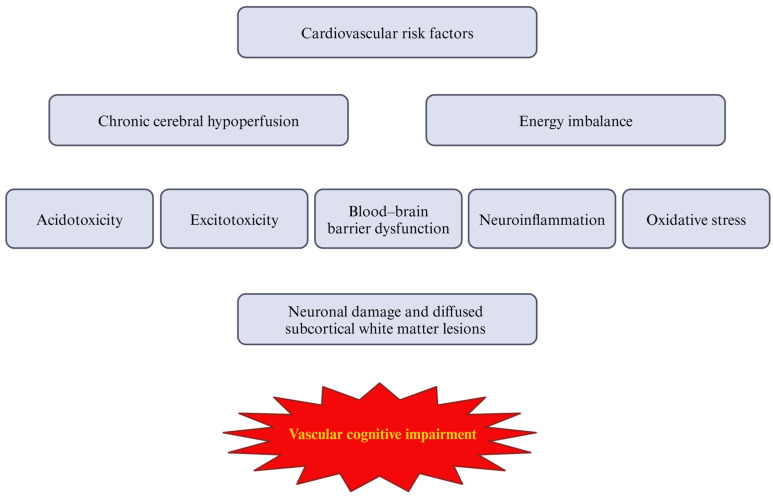
The sequence of vascular cognitive impairment pathophysiology.

VCI is a progressive disorder without a registered causal treatment; however, there are some preventive options affecting traditional risk factors for the disease [9]. In addition, no specific biomarker has been established to date, so earlier diagnostic tools are needed [9]. Typical clinical manifestations of VCI involve problems with reasoning, planning, judgment, and cognitive decline, particularly in executive function, which may be accompanied by gait disturbances [10]. However, VCI encompasses a broad spectrum of cognitive deficits, ranging from milder forms, such as vascular mild cognitive impairment (VaMCI), to more severe conditions like vascular dementia (VaD) [11]. According to the National Institute of Neurological Disorders and Stroke–Canadian Stroke Network, the term VCI applies to cognitive deficits resulting from or related to underlying vascular factors, regardless of whether the pathogenesis of the vascular lesion is ischemic or hemorrhagic [12]. Therefore, the classification of VCI is complex and often ambiguous. The Vascular Impairment of Cognition Classification Consensus Study (VICCCS) has identified four basic subtypes of dementia: post-stroke dementia, multi-infarct (cortical) dementia, subcortical ischemic VCI, and mixed dementias, which encompass varying combinations of two or more etiologic cognitive deficits [13]. The presence of vascular abnormalities, leading to chronic cerebral hypoperfusion and subsequent hypoxia, is a common feature of these pathologies. According to Rajeev et al., chronic cerebral hypoperfusion appears to be a critical underlying mechanism that integrates various factors, resulting in ischemia-induced neuronal damage [14]. Thus, the central nervous system (CNS) is particularly sensitive to disruptions in blood supply, and normal perfusion is essential for maintaining neuronal activity. Neuronal function and survival, as well as the structural and functional integrity of the brain, are critically dependent on a constant supply of oxygen and nutrients [15]. Therefore, chronic cerebral hypoperfusion and ischemia are associated with omnidirectional changes within the CNS, promoting oxidative stress, inflammatory processes, and subsequent neuronal damage. Together, these pathological changes contribute to the development of VCI (summarized in Table 1 and Figure 1) [16]. Finally, given the extensive research on the gut microbiota in various inflammation-mediated conditions, it is important to consider its potential role in the development of VCI. Recognizing the fact that the comprehension of pathological processes underlying VCI has evolved in the last decades, this narrative review aims to summarize the current understanding of the pathogenesis of VCI, focusing on the key role of blood–brain barrier (BBB) dysfunction, oxidative stress, and immunological processes in the pathophysiology of the disease. Furthermore, the potential influence of the gut microbiota on the development of VCI will be explored. To date, the vast majority of papers have focused on detailed descriptions of separated processes, without providing a coherent synthesis of all these elements, particularly the link between the gut microbiota and the description of disease pathophysiology.

## 2. Materials and Methods

To conduct a comprehensive analysis of the topic, a thorough search of the relevant literature was undertaken using the PubMed and Google Scholar databases, covering studies from their inception to 23 September 2024. Both experimental and clinical studies were included. Titles and abstracts were screened for key terms such as ‘vascular cognitive impairment’, ‘vascular dementia’, ‘molecular mechanism’, ‘molecular basis’, and ‘gut microbiota’. To ensure thorough coverage, relevant references from the identified articles were also manually reviewed by the authors.

## 3. Molecular Basis of Vascular Cognitive Impairment

**Figure 2:**
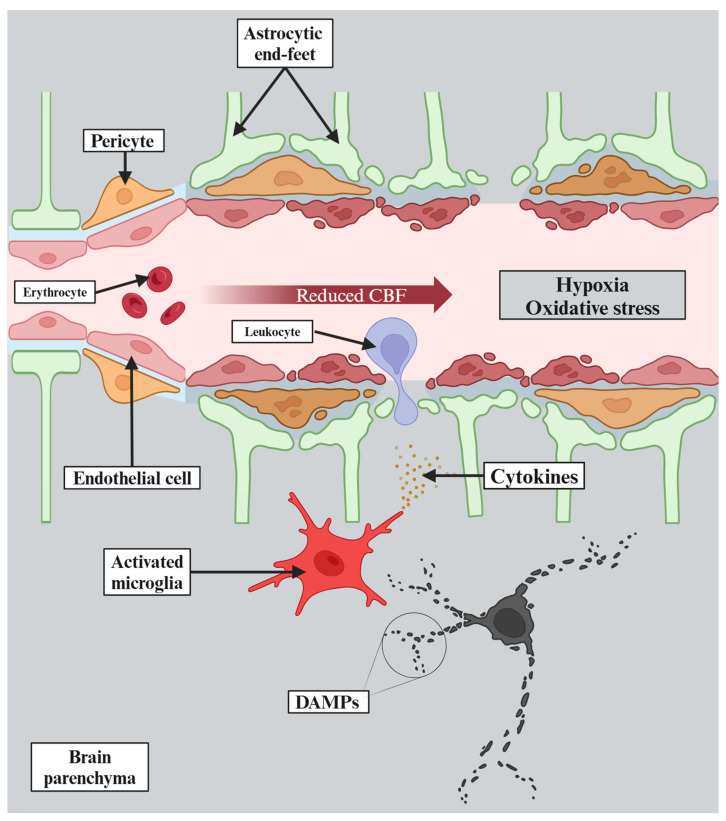
Chronic cerebral hypoperfusion leads to reduced cerebral blood flow (CBF), which then causes hypoxia. Moreover, hypoxia results in rapid depletion of adenosine triphosphate that triggers excitotoxicity and acidotoxicity. The pathophysiological changes described above lead to oxidative stress and further cellular damage. Thereafter, damage-associated molecular patterns (DAMPs) are released, triggering activation of microglia, which consequently release pro-inflammatory cytokines that exacerbate neuroinflammation. Finally, all of this causes endothelial dysfunction resulting in blood–brain barrier dysfunction and further deterioration of the whole process.

VCI comprises a heterogeneous group of cognitive deficits resulting from various vascular pathologies. It can result from a widespread acute ischemic stroke affecting strategic areas of the brain, such as the frontal cortex, or from multiple cortical infarcts causing direct damage to cerebral tissue [17]. On the other hand, diffuse subcortical ischemic damage associated with cerebral small vessel disease (CSVD) is also a common cause of VCI. This is why it is so difficult not only to classify these pathologies, but also to understand their molecular mechanisms [13]. Yet, it is known that chronic cerebral hypoperfusion and thromboembolic events, which reduce cerebral blood flow and induce hypoxia, cause oxidative stress and inflammation in the nervous system [18]. The molecular mechanisms underlying the consequences of chronic cerebral hypoperfusion are summarized in Figure 2.

### 3.1. Energy Imbalance, Excitotoxicity, and Acidotoxicity

Although the brain accounts for less than 2% of total body weight, it requires over 20% of the body’s energy supply [19]. Hence, it is not surprising that a blood flow of at least 50 mL/100 g/min is required to provide an adequate supply of both glucose and oxygen [20]. Consequently, it is relatively easy to induce damage through chronic cerebral hypoperfusion. Under hypoxic–ischemic conditions, neurons, which derive their energy primarily from aerobic glucose metabolism, rapidly become depleted of adenosine triphosphate (ATP). This condition impairs the function of ATP-dependent ion pumps, including ATP-dependent sodium–potassium pumps (Na^+^/K^+^-ATPases), which are responsible for maintaining the resting potential of cells [14]. As a result, potassium ions (K^+^) flow out of the cell while sodium ions (Na^+^) flow in the opposite direction, leading to anoxic depolarization of not only neurons but also of glial cells. This results in an electrochemical imbalance and the opening of voltage-gated calcium (Ca^2+^) channels in the presynaptic membrane, causing an excessive influx of Ca^2+^ ions that cannot be efficiently removed from the cell [20]. The elevated intracellular Ca^2+^ levels, in turn, induce an unrestricted release of glutamate, the primary excitatory neurotransmitter in the CNS, into the synaptic cleft [20,21].

Also, ATP deficiency impairs glutamate transporters, resulting in insufficient reuptake of glutamate by astrocytes [20]. A study by Ketheeswaranathan et al. using a mouse model of ischemic stroke induced by middle cerebral artery occlusion demonstrated that the mRNA expression of glial cell glutamate transporters was significantly reduced in the hippocampus and cerebral cortex of ischemic stroke mice compared to the control group [22]. Combined, these factors cause an energy imbalance that leads to excessive glutamate accumulation in the synaptic cleft, ultimately inducing a state of excitotoxicity within the CNS [23].

Excitotoxicity, in general, refers to cell death caused by the overactivity of excitatory amino acids. In the case of neuronal excitotoxicity, it is specifically glutamate-induced neuronal damage. Through its interaction with N-methyl-D-aspartate (NMDA) receptors, glutamate may trigger an additional influx of Ca^2+^ ions into neurons, causing further release of glutamate [24]. The interaction of glutamate with certain alpha-amino-3-hydroxy-5-methyl-4-isooxazole-propionic acid (AMPA) receptors, long thought to be impermeable to calcium, may also increase intracellular Ca^2+^ concentration. In addition, the expression of calcium-permeable AMPA receptors may be increased in pathological conditions. Since AMPA receptors are permeable to zinc cations, which induce mitochondrial damage, their role in excitotoxicity should not be underestimated [25]. Studies also report glutamate-independent pathways of Ca^2+^ influx, such as the melastatin subfamily of transient receptor potential channels or acid-sensing ion channels (ASICs) [26]. Given that hypoxia favors anaerobic glucose metabolism, it ultimately leads to the reduction of pyruvate to lactate anions, which is accompanied by the production of protons (H^+^) causing acidosis [27]. Over the years, various theories have been proposed as to how acidosis causes neural damage. The current understanding of acidotoxicity emphasizes the role of ASICs, which are activated by a decrease in extracellular pH, enabling the influx of Ca^2+^ ions into neurons [27,28]. Additionally, proton-activated chloride (Cl^−^) channels also trigger Cl^−^ influx into neurons [27]. The disruption of ion balance, leading to the accumulation of Na^+^, Cl^−^, and Ca^2+^ ions in neurons, is accompanied by an osmotic influx of water, resulting in cytotoxic edema and ultimately oncosis [29,30]. Another potential mechanism of Ca^2+^ influx-driven cell death may be that of the activation of catabolic enzymes, such as endonuclease and calpain, leading to apoptosis or necrosis depending on the metabolic state of the neuron [20,31].

### 3.2. Oxidative Stress

Reactive oxygen species (ROS), or free radicals, are formed during oxidative phosphorylation in the inner mitochondrial membrane, a process essential for the survival and function of all cell types [32]. ROS, including superoxide anions, peroxide, hydroxyl radicals, and singlet oxygen, contribute to various physiological processes such as cellular communication, modulation of gene expression, and immune response [33]. However, excessive ROS production, beyond the neutralizing capacity of the CNS antioxidant defenses, can lead to oxidative damage, including extensive lipid peroxidation, but also to protein and DNA damage. This phenomenon is known as oxidative stress and has been linked to numerous neurodegenerative disorders [32]. Even though the hypothesis that ROS may be involved in the aging process dates back to the 1950s, for many years, little was known about the molecular basis of this association [34]. In fact, the level of oxidative stress is still difficult to measure directly due to the short lifespan and rapid reactivity of ROS. Therefore, studies often use proxies, mainly the products of lipid peroxidation and damaged proteins or DNA [35]. Given that the CNS is mainly composed of lipid-rich tissues and that fatty acids are particularly susceptible to peroxidation, oxidative stress is naturally associated with neurological diseases, including AD and VCI [36].

A study by Gustaw-Rothenberg et al. revealed elevated markers of oxidative stress, including malondialdehyde (MDA), a product of lipid peroxidation, in both AD and VaD groups, compared to healthy controls. Also, patients with VaD had significantly higher plasma concentrations of oxidative stress markers than those with AD [37]. Based on animal studies, oxidative stress is considered a key component of ischemic brain injury [38], a finding further confirmed by Kelly et al. Their study reported elevated levels of F2-isoprostanes (F2IPs), another lipid peroxidation marker, in early stroke patients compared to controls, which correlated with plasma matrix metalloproteinase-9 (MMP-9) levels in all stroke patients [39]. Thus, metalloproteinases may be responsible for the proteolytic dysfunction of the BBB and the white matter hyperintensities characteristic of VCI [40].

Furthermore, recent studies have shown that the levels of antioxidants may decrease during chronic cerebral hypoperfusion [38]. As mentioned above, chronic cerebral hypoperfusion leads to Ca^2+^ imbalance, which subsequently causes an overproduction of ROS in various pathways, including those involving the nicotinamide adenine dinucleotide phosphate oxidase (Nox) family and nitric oxide synthase (NOS) [14]. Based on animal model studies, Nox might be a key contributor to chronic cerebral hypoperfusion-induced oxidative stress and cognitive dysfunction [38]. For instance, a study conducted by Choi et al. in a rat model of chronic cerebral hypoperfusion, induced by bilateral common carotid artery ligation (two-vessel occlusion, 2VO), showed increased MDA levels and increased Nox1 expression in the hippocampi of 2VO rats. The study also demonstrated that Nox1 inhibition by apocynin significantly reduced superoxide production and oxidative DNA damage, as assessed by 8-hydroxy-2′-deoxyguanosine immunoreactivity. Additionally, the neuronal loss in the CA1 hippocampal subfield, observed in 2VO rats, was significantly reduced following apocynin administration. Furthermore, apocynin treatment resulted in shorter mean session latencies and fewer search errors in spatial memory tests compared to the 2VO group [41].

NO-mediated vasodilation is another mechanism affected by oxidative stress. Since superoxide reacts with NO much faster than it can be neutralized by superoxide dismutases (SODs), the availability of NO produced by endothelial NO synthase (eNOS) is reduced. Thus, oxidative stress not only impairs endothelial function, leading to reduced cerebral blood flow, but it also disrupts neuronal signaling and neurogenesis, ultimately contributing to VCI [42]. Furthermore, ROS are produced not only during hypoxia; reperfusion and reoxygenation can also trigger their extensive release [33,43]. During hypoxia, electrons with reduction potential accumulate in the mitochondria. Upon the restoration of oxygenation, excess electrons can escape from the mitochondrial electron transport chain and interact with oxygen molecules to generate ROS [43].

Finally, oxidative stress contributes to inflammation, causing further damage. ROS can also cause intracellular damage starting in the mitochondria. Subsequent mitochondrial dysfunction results in the release of mitochondrial-derived damage-associated molecular patterns (DAMPs), typically ROS-modified mitochondrial DNA and proteins [43]. Mitochondrial-derived DAMPs can activate the endogenous pathway of neuroinflammation through the NLRP3 inflammasome, causing activation of pro-caspase-1 and subsequent release of interleukin (IL)–18 and IL–1β. Also, extracellular mitochondrial-derived DAMPs activate a toll-like receptor 9 (TLR9)-dependent inflammatory cascade in other glial cells [44]. This creates a self-perpetuating vicious cycle that further accelerates neurodegeneration [43].

### 3.3. Neuroinflammation

The CNS has long been considered an immune-privileged area [45]. However, this concept has changed dramatically since the early days of pioneering neuroimmunological research [46]. The current understanding of CNS–immune interactions has not only revealed communication between the CNS and the peripheral immune system, but it has also identified the presence of a functional meningeal lymphatic system within the CNS [47]. Therefore, neuroinflammation, which can be defined as an immune response in the CNS, is not a spatially separated process as peripheral leukocytes can infiltrate the CNS, drawn in by secreted cytokines [48]. However, neuroinflammation is mainly regulated by microglia, CNS-residing immune cells, which originate from macrophage precursors [48]. Neuroinflammation itself is a complex process that contributes to both ischemic brain injury and also brain tissue regeneration [20]. While the classification of neurological disorders includes certain conditions categorized as inflammatory, e.g., multiple sclerosis, recent studies suggest that inflammation is associated with other diseases not traditionally considered neuroinflammatory, including Alzheimer’s disease [49].

A meta-analysis conducted by Custodero et al. demonstrated that serum or plasma IL–6 and tumor necrosis factor (TNF)–α levels were significantly elevated in patients with VaD compared to healthy controls, but only IL–6 levels differed significantly between VaD and AD patients. C-reactive protein (CRP) levels were not significantly altered in patients with VaD compared to controls [50]. Additionally, serum IL–6 concentration is associated with white matter hyperintensities, the hallmark of VCI, as demonstrated by Kumiko Nagai et al. [51]. Furthermore, a bidirectional Mendelian randomization study by Yuge Xia et al. demonstrated an association between VaD and seven circulating cytokines, including IL–7, IL–17, and interferon gamma (IFN)–γ [52]. Therefore, it seems reasonable to consider neuroinflammatory components as integral to the VCI pathophysiology [16,53].

Furthermore, studies using a rat model of CCH, 2VO, have highlighted the activation of both astrocytes and microglia in response to hypoxia and ischemia [54]. Based on histopathologic examination of brain tissue from five patients with VaD, Rosenberg et al. suggest that microglia-/macrophage-induced damage may be associated with the progressive form of VaD [40]. Microglia are the first line of immune response within the CNS, recognizing pathogen-associated molecular patterns (PAMPs) and DAMPs [55]. As described above, during chronic cerebral hypoperfusion, damaged cells release cellular debris, including fragments such as mitochondrial DNA, and act as DAMPs that activate microglia [44].

In general, microglia adopt two phenotypic states: pro-inflammatory (M1) and anti-inflammatory (M2). Activation by DAMPs through TLR4 induces the M1 phenotype [56]. A study in hypoxic neonatal rats revealed increased post-hypoxia TLR4 immunofluorescence and expression. Furthermore, TLR4 activation caused a significant increase in TNF–α and IL–1β expression, which was suppressed by TLR4 neutralization with a hypoxia-inducible factor (HIF)-1α antibody prior to exposure to hypoxia [57]. However, hypoxia itself causes upregulation of HIF-1α, which is a nuclear transcription factor that activates nuclear factor (NF)-κB [16]. NF-κB is considered to be one of major mediators of the release of pro-inflammatory cytokines, including TNF–α, IL–1β, and IL–6 [58]. The release of these cytokines can lead to cell death and neuronal loss, which exacerbate inflammation in the nervous system and can manifest clinically as cognitive decline [43]. Additionally, microglia can produce adhesion molecules and chemokines [14]. IL–1β and TNF–α have also been shown to influence BBB permeability [59]. This cascade causes the recruitment of leukocytes and facilitates their infiltration into the CNS [14,20]. These leukocytes secrete further pro-inflammatory cytokines and MMPs [20], which are also produced and released by microglia [16]. Not only do MMPs cause damage to the extracellular matrix, but they also proteolyze the cerebrovascular basement membrane and tight junctions (TJs), thereby dysregulating BBB integrity [60]. Thus, the above processes contribute to both neurodegeneration and further exacerbation of inflammation.

### 3.4. Blood–Brain Barrier Dysfunction

The BBB, together with the blood–cerebrospinal fluid barrier at the choroid plexus and the arachnoid barrier, is a key component of the system that isolates the CNS from the peripheral systemic circulation. The BBB is a selectively permeable structure that separates the systemic blood circulation from the brain parenchyma and controls the influx of both nutrients and potentially toxic substances, as well as the outflow of CNS-derived metabolites. The BBB is not a homogeneous structure, as its composition and permeability vary depending on the specific region of the CNS it encloses [61]. It is composed of endothelial cells interconnected by TJs, together with pericytes and astrocyte end-feet that collectively regulate barrier integrity and function [62].

Recent studies have identified the dysregulation of the BBB in various pathological conditions, including cognitive disorders [63]. BBB dysfunction has also been observed in animal models of chronic cerebral hypoperfusion [64]. For instance, Ueno et al. conducted a study using a 2VO rat model of chronic cerebral hypoperfusion to evaluate BBB permeability. They used horseradish peroxidase (HRP), an exogenous tracer often used to assess BBB integrity. Their findings demonstrated that chronic cerebral hypoperfusion increased BBB permeability, as indicated by the presence of HRP in the brain parenchyma, a phenomenon absent in the control group [65]. Similar results have been reported in clinical studies. Wallin et al. showed that patients with VaD exhibited significantly increased cerebrospinal fluid to serum albumin ratios compared to the control group, indicating compromised BBB integrity. They also suggest that the observed alterations in BBB permeability are more likely a consequence of underlying vascular dysfunction rather than a direct effect of stroke episodes [66].

In support of this theory, several traditional cardiovascular risk factors, including hypertension, hypercholesterolemia, diabetes, and smoking, are known to induce endothelial dysfunction. This results in decreased levels of vasodilators, such as nitric oxide (NO), and increased production of endothelium-derived vasoconstricting factors [67]. Hence, these pathologies could disrupt the regulation of cerebral blood flow, potentially leading to hypoperfusion and subsequent hypoxia [68]. Chronic cerebral hypoperfusion could affect the expression of TJs in endothelial cells, resulting in increased BBB permeability. Also, NMDA receptors are present on various brain cell types, including BBB endothelial cells. During chronic cerebral hypoperfusion-induced excitotoxicity, prolonged activation of NMDA receptors on endothelial cells initiates a cascade similar to that observed in neurons, ultimately contributing to the disruption of BBB integrity. The increased permeability of the BBB allows infiltration by immune cells, which exacerbate BBB dysfunction by promoting OS and neuroinflammatory processes [23].

## 4. The Role of Gut Microbiota in Vascular Cognitive Impairment

The role of the microbiota has been widely studied since its discovery, with the gut microbiota being of particular interest to neuroscientists. This attention has increased even further with the discovery of the gut–brain axis and the recognition of these bidirectional interactions in health and disease [69]. The healthy intestinal microbiota is predominantly composed of two phyla, Firmicutes and Bacteroidetes, which together account for 90% of all bacteria. The Firmicutes phylum consists of over 200 genera, 95% of which are *Clostridium* genera. The Bacteroidetes phylum, on the contrary, consists mainly of *Bacteroides* and *Prevotella* genera [70]. Dysbiosis, which is an imbalance in the Firmicutes/Bacteroidetes ratio, is often associated with inflammatory disorders [71,72].

Dysbiosis has also been shown to occur in the course of cognitive impairment [73]. Unfortunately, most of the studies analyzing the effect of gut microbiota on cognitive performance were conducted in patients with dementia in general or focused on patients with Alzheimer’s disease. For instance, a study by Kan et al. demonstrated diminished amounts of the *Ruminococcus*, *Butyricimonas*, and *Oxalobacter* genera in MCI patients compared to controls. These genera could discriminate MCI patients from the controls and were linked to attention and executive function. However, according to this study, there was no significant difference in alpha and beta diversity between the two groups [74]. In contrast, Vogt et al. identified changes in bacterial abundance in the gut microbiota of AD patients, including a decrease in Firmicutes and an increase in Bacteroidetes phyla, and a decrease in the *Bifidobacterium* genus [75]. There is also growing evidence that the gut microbiota may influence the development of VCI [76]. In a study by Fongang et al., the *Barnesiella intestinihominis* bacterium was associated with markers indicating greater progression of CSVD, one of the subtypes of VCI. In contrast, the abundance of *Pseudobutyrivibrio* and *Ruminococcus* genera was associated with decreased markers of CSVD [77].

**Figure 3:**
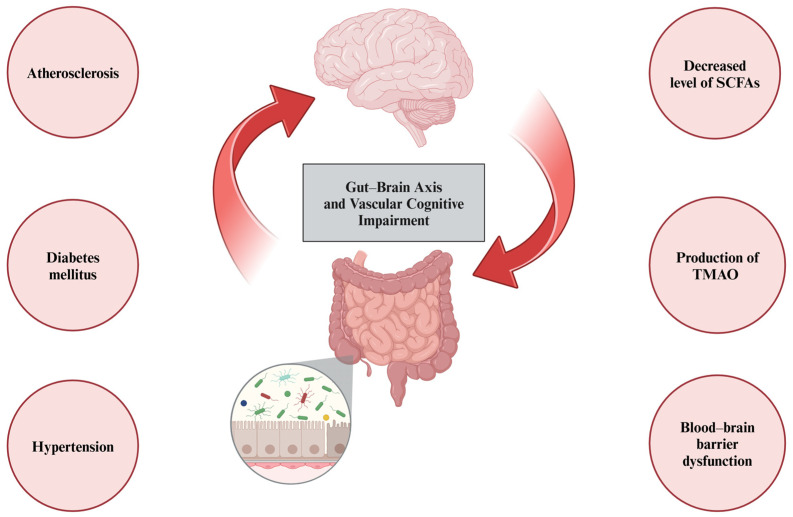
Gut microbiota may influence vascular cognitive impairment omnidirectionally. First, its role has been established in traditional vascular risk factors, such as atherosclerosis, diabetes mellitus, or hypertension. Second, dysbiosis leads to reduced levels of short-chain fatty acids (SCFAs) and elevated levels of trimethylamine N-oxide (TMAO), leading to blood–brain barrier dysfunction and chronic low-grade inflammatory processes.

Furthermore, the role of gut microbiota has been highlighted in the pathogenesis of hypertension [78], diabetes mellitus [79], cholesterol metabolism disorders, and atherosclerosis [80], which are traditional cardiovascular risk factors that may contribute to VCI [81]. Finally, the microbiota–gut–brain axis has been implicated in chronic cerebral hypoperfusion [82]. A study conducted in an animal model of chronic cerebral hypoperfusion revealed that 2VO rats exhibited elevated levels of *Bacteroidetes* and *Verrucomicrobia* genera and a decreased abundance of *Firmicutes* and *Tenericutes*. In addition, a quantitative analysis of short-chain fatty acid (SCFA)-producing bacteria, including the *Prevotellaceae* family and the *Bifidobacterium* genus, showed their lower levels in the 2VO group [82]. SCFAs, such as acetate, propionate, and butyrate, are primary metabolites synthesized in the colon by the gut microbiota through the fermentation of dietary fiber. They are thought to be pivotal in the gut–brain axis crosstalk, as they have anti-inflammatory properties, increase BBB integrity, and enhance neurogenesis [83]. Additionally, research conducted in the 2VO-induced chronic cerebral hypoperfusion rat model showed that intraperitoneal butyrate administration improved cognitive function, as measured by spatial learning and memory tests [84]. Another gut microbiota-derived metabolite, trimethylamine N-oxide (TMAO) is a proatherogenic and prothrombotic factor, implicated in the pathogenesis of cardiovascular disorders [85]. A study by Deng et al. showed that the administration of TMAO aggravates VCI in 2VO rats. In addition, TMAO activates the NLRP3 inflammasome, which activates pro-caspase-1, and decreases the expression of silent information regulator 1 (SIRT1). Therefore, TMAO is thought to exacerbate oxidative stress, neuroinflammation, and ultimately apoptosis [86]. However, human-based research is needed to determine the exact impact of gut microbiota and their metabolites on VCI. All the multidirectional effects of gut microbiota on VCI are summarized in Figure 3. The gut microbiota is not only related to the pathophysiology of VCI, but also associated with its coexistence with celiac disease [87]. A majority of studies pertaining to celiac disease highlighted the significance of intestinal dysbiosis in the course of the disease [88]. Additionally, a study by Pennisi et al. using Transcranial Magnetic Stimulation revealed that patients with celiac disease had enhanced intracortical facilitation [89]. According to Bella et al., patients with VCI also had enhanced intracortical facilitation at baseline assessment; however, it showed a trend to decrease during a follow-up of approximately 2 years, compared with control group [90]. Therefore, Transcranial Magnetic Stimulation would be a useful diagnostic tool in both celiac disease and VCI. Finally, diet might influence cognitive functions directly or by affecting gut microbiota [91]. According to Jennings et al., some diet components, such as omega-3 fatty acids, can improve cognitive functions [92]. Diets rich in fiber and omega-3 fatty acids increase SCFA production and the amount of SCFA-producing bacteria, subsequently exerting an anti-inflammatory effect [93]. Moreover, Pennisi et al. indicated that daily mocha coffee and red wine consumption might be synergistically associated with global cognition and mood status in patients with VaMCI [94].

## 5. Limitations and Future Research Directions

This article addresses a broad and complex topic. Additionally, little is still known about the exact sequence of pathological changes in the course of VCI, as many studies focus on a particular element of disease pathophysiology rather than the process as a whole. Also, we are aware that a still relatively small research base of studies analyzing the role of gut microbiota in VCI pathogenesis is a limitation of our paper. Moreover, huge prospective longitudinal studies describing changes in the gut microbiota with the progression of the disorder are lacking, and, to date, there have been no multicenter, randomized clinical trials evaluating the targeting of the gut microbiota as a treatment option for VCI. Therefore, clinical studies are needed to determine this relationship and, potentially, to implement a causal treatment for this progressive disorder. Also, research directly linking the molecular basis of disease to clinical practice is warranted [95], as there is still no specific biomarker for VCI [9].

## 6. Conclusions

Although VCI is becoming a major healthcare problem worldwide, little is still known about its pathophysiology. Chronic cerebral hypoperfusion is its main driver, which subsequently triggers a whole cascade of molecular changes, including an energy imbalance, leading to glutamate excitotoxicity, acidotoxicity, and oxidative stress. All of the above trigger the activation of microglia and the release of pro-inflammatory cytokines that recruit systemic immune cells and lead to their infiltration into the central nervous system, resulting in neuroinflammation. BBB dysfunction also plays a critical role in the pathological process. However, it is difficult to determine whether it is the first element of the cascade or an effect that further exacerbates neuroinflammation. In particular, little is known about the role of gut microbiota in VCI development and progression. Given that there is no pharmacological treatment for VCI to date [96], targeting gut microbiota is a novel and promising future option for clinicians, thus underscoring the translational nature of the research analyzing these interrelationships.

## Figures and Tables

**Table 1:** Molecular mechanisms underlying vascular cognitive impairment, their clinical manifestations, and potential therapeutic implications.

Molecular Mechanism	Description of the Process	Clinical Manifestation	Potential Treatment Approaches
Chronic Cerebral Hypoperfusion	Reduced blood flow causes hypoxia, which leads to an energy imbalance and subsequent excitotoxicity due to excess glutamate release	Diffused subcortical white matter lesions observed in magnetic resonance imaging	Controlling the risk factors, such as hypertension, dyslipidemia, and diabetes mellitus
Oxidative Stress	Overproduction of reactive oxygen species that damage cellular components, leading to cell death	Widespread neuronal and synaptic damage contributes to cognitive decline	Antioxidant therapy and mitochondrial improvement
Neuroinflammation	Chronic activation of microglia and release of pro-inflammatory cytokines with further infiltration of the central nervous system by peripheral immune cells	Prolonged inflammation leading to neuronal damage and loss, manifesting clinically as progressive cognitive and functional impairment	Anti-inflammatory agents and immunomodulators
Blood–Brain Barrier Dysfunction	Increased permeability of the blood–brain barrier enabling inflow of neurotoxic substances and peripheral immune cells into the brain parenchyma	Cerebral edema and diffused subcortical white matter lesions, which can manifest as cognitive decline	Controlling cardiovascular risk factors, development of novel agents decreasing blood–brain barrier permeability

## Data Availability

Not applicable.
